# Serine Phosphorylation of the Hepatitis C Virus NS5A Protein Controls the Establishment of Replication Complexes

**DOI:** 10.1128/JVI.02995-14

**Published:** 2014-12-31

**Authors:** Douglas Ross-Thriepland, Jamel Mankouri, Mark Harris

**Affiliations:** School of Molecular and Cellular Biology, Faculty of Biological Sciences, and Astbury Centre for Structural Molecular Biology, University of Leeds, Leeds, United Kingdom

## Abstract

The hepatitis C virus (HCV) nonstructural 5A (NS5A) protein is highly phosphorylated and involved in both virus genome replication and virion assembly. We and others have identified serine 225 in NS5A to be a phosphorylation site, but the function of this posttranslational modification in the virus life cycle remains obscure. Here we describe the phenotype of mutants with mutations at serine 225; this residue was mutated to either alanine (S225A; phosphoablatant) or aspartic acid (S225D; phosphomimetic) in the context of both the JFH-1 cell culture infectious virus and a corresponding subgenomic replicon. The S225A mutant exhibited a 10-fold reduction in genome replication, whereas the S225D mutant replicated like the wild type. By confocal microscopy, we show that, in the case of the S225A mutant, the replication phenotype correlated with an altered subcellular distribution of NS5A. This phenotype was shared by viruses with other mutations in the low-complexity sequence I (LCS I), namely, S229D, S232A, and S235D, but not by viruses with mutations that caused a comparable replication defect that mapped to domain II of NS5A (P315A, L321A). Together with other components of the genome replication complex (NS3, double-stranded RNA, and cellular lipids, including phosphatidylinositol 4-phosphate), the mutation in NS5A was restricted to a perinuclear region. This phenotype was not due to cell confluence or another environmental factor and could be partially transcomplemented by wild-type NS5A. We propose that serine phosphorylation within LCS I may regulate the assembly of an active genome replication complex.

**IMPORTANCE** The mechanisms by which hepatitis C virus replicates its RNA genome remain poorly characterized. We show here that phosphorylation of the viral nonstructural protein NS5A at serine residues is important for the efficient assembly of a complex that is able to replicate the viral genome. This research implicates cellular protein kinases in the control of virus replication and highlights the need to further understand the interplay between the virus and the host cell in order to develop potential avenues for future antiviral therapy.

## INTRODUCTION

Hepatitis C virus (HCV) currently infects an estimated 170 million individuals worldwide and in 85% of cases establishes a chronic infection, which typically progresses to liver cirrhosis and hepatocellular carcinoma (1). The virus has a single-stranded RNA genome that codes for an ∼3,000-amino-acid polyprotein that is cleaved co- and posttranslationally into 10 mature viral proteins: the core and the E1 and E2 envelope proteins, which make up the viral particle, followed by the viroporin p7 and the nonstructural 2 (NS2) protein, which possesses autoprotease activity. The remaining nonstructural (NS) proteins, NS3, NS4A, NS4B, NS5A, and NS5B, are necessary and sufficient for genome replication (2). With the advent of the full-length clone of a genotype 2a isolate (JFH-1) able to undergo the complete virus life cycle in cell culture, significant progress has been made in understanding how the different viral proteins contribute to the processes of genome replication and virus assembly. In this regard, NS5A has been shown to play a critical role in both of these processes and is known to perturb numerous host pathways in favor of virus persistence.

The 5′ untranslated region (UTR) of the viral genome contains an internal ribosome entry sequence (IRES) that allows viral proteins to be translated in a cap-independent fashion immediately after the viral genome is delivered into the cytoplasm. HCV proteins then rapidly recruit and remodel endoplasmic reticulum-derived membranes to form a membranous web (MW), a subcellular structure comprised of single-membrane, double-membrane, and multimembrane vesicles (SMVs, DMVs, and MMVs, respectively) that are enriched in viral (e.g., NS3, NS5A, and NS5B) and host cell (e.g., VAP-A) proteins (3, 4). By analogy to other positive-sense RNA viruses, this enrichment of viral NS proteins, the presence of double-stranded RNA (dsRNA), and the observation that isolated membrane fractions retain replication activity have led to the proposal that the MW is the site of viral genome replication (3). In Huh7 cells, the MW is extensively distributed throughout the cytoplasm, correlating with the observed subcellular distribution of the NS5A and NS3 proteins as discrete puncta throughout the cytoplasm. The formation and maintenance of the MW have been shown to be dependent on the activation of the phosphatidylinositol 4-kinase (PI4K) type III alpha (PI4KIIIα) isoform by NS5A and the subsequent elevated production of phosphatidylinositol 4-phosphate (PI4P) by this lipid kinase (5–7). Additionally, lipid droplets (LDs) are a host organelle responsible for the normal cellular storage of neutral lipids and have been shown to be central to the HCV life cycle. While the site of virus assembly has not been elucidated, it is known that both the core and NS5A proteins coat the surface of LDs, displacing the host factor adipocyte differentiation-related protein (ADRP). In order for virus genomes that have been newly synthesized in the MW to be packaged into virions, the core protein-coated LDs are thought to be recruited to the MW, possibly in an NS5A-directed manner, in order to allow the encapsidation of viral RNA (8, 9).

NS5A is a highly phosphorylated protein comprised of three domains and tethered to membranes by an N-terminal amphipathic helix. Domain I is highly structured and sufficient for NS5A to dimerize, while domains II and III are intrinsically disordered but have recently been shown to have elements of transient secondary structure throughout (10–12). These three domains are linked by low-complexity sequences (LCSs) (13); a serine-rich sequence (LCS I) links the first two domains, while a proline-rich sequence (LCS II) links the last two domains. NS5A exists in two different phosphorylated forms that can be readily resolved by SDS-PAGE. These forms, termed the basal phosphorylated and hyperphosphorylated species, are thought to represent different functional pools of NS5A involved in different stages of the virus life cycle, but as yet there is little direct evidence to support this hypothesis. Until recently, the sites of NS5A phosphorylation had not been unambiguously mapped, their locations having been inferred from mutagenesis data. Recently, however, we and others have used mass spectrometry to demonstrate that NS5A, purified from cells actively replicating the HCV subgenomic replicon (SGR), is extensively phosphorylated within LCS I, containing at a minimum seven phosphorylation sites (14–16). We also showed that NS5A contains phosphorylation sites in domain I, domain II, and LCS II. Previous work to characterize the role of LCS I phosphorylation has focused almost entirely on a role in virus replication and/or assembly/release, and numerous studies have shown several serines to be important for viral replication, but in a genotype-dependent manner. These studies have, however, not explored the mechanism through which the phosphorylation of LCS I might be acting.

We show here that the phosphorylation of serine 225 within LCS I is critical for the correct localization of viral proteins during infection. Ablation of serine 225 phosphorylation results in a 10-fold reduction in replicative fitness. We show using confocal microscopy that this phenotype is concomitant with a dramatic relocalization of both cellular and viral factors known to participate in genome replication. This phenotype is shared with mutants with other serine mutations in LCS I, further illustrating the complexity of phosphorylation in the region of NS5A.

## MATERIALS AND METHODS

### Plasmids.

DNA constructs of and the full-length pJFH-1 virus (17) and the luciferase subgenomic replicon (SGR-luc-JFH-1) (18) were used throughout the study. Previously, the unique restriction sites flanking NS5A, BamHI and AfeI, were introduced into both the full-length virus and SGR-luc-JFH-1 constructs; these constructs are denoted mJFH-1 and mSGR-luc-JFH-1, respectively, and have shown been to have no effect on virus genome replication or assembly and release (19). Site-directed mutagenesis of NS5A was performed with a QuikChange kit (Stratagene) and a LITMUS28i (NEB) subclone containing an NsiI/HindIII fragment of the mJFH-1 cDNA. Then, mutant NS5A was cloned into either mSGR-luc-JFH-1 or mJFH-1 via flanking BamHI/AfeI sites, and correct insertion was confirmed by sequencing. For the insertion of the SNAP and CLIP tags into NS5A, the gene was amplified from the pSNAPf and pCLIPf vectors (NEB) using primers that insert a flexible linker sequence (GSSGSS) on either side of the insertion (primer sequences are available upon request). This amplicon was inserted into the aforementioned LITMUS28i subclone by BclI digestion, and correct insertion was verified by sequencing. The NS5A-SNAP or NS5A-CLIP region was then inserted into mSGR-luc-JFH-1 via flanking RsrII/AfeI sites. For plasmid expression, mutated NS5A-coding sequences were subcloned into the pCMV(NS3-5B) vector as described previously (20). All mutations were verified by sequencing.

### Cell culture.

Huh7 cells were cultured in Dulbecco's modified Eagle's medium (DMEM; Sigma) supplemented with 10% fetal bovine serum (FBS), 100 IU penicillin/ml, 100 μg streptomycin/ml, and 1% nonessential amino acids in a humidified incubator at 37°C with 5% CO_2_.

### Electroporation of replicon and virus RNA constructs.

The preparation of *in vitro* transcripts and electroporations for both mSGR-luc-JFH-1 and mJFH-1 were conducted as described previously (20). In brief, 2 × 10^6^ Huh7 cells in diethyl pyrocarbonate (DEPC)–phosphate-buffered saline (PBS) were electroporated with 5 μg of *in vitro* RNA transcripts using a square-wave protocol at 260 V for 25 ms. Subsequently, cells were resuspended in complete DMEM and seeded at 1 × 10^4^ cells/cm^2^ culture area in either 12- or 6-well dishes.

### Virus titration.

Supernatants were titrated on Huh7 cells in a 96-well format as follows. Clarified virus supernatant was serially diluted 2-fold in complete DMEM before addition of 100 μl to cells that had been seeded 8 h earlier at 8 × 10^3^ cells/well (final volume, 200 μl). The counting of infected cells was automated by acquisition and analysis using an IncuCyte ZOOM platform. The virus titer was determined by averaging the positive cell count from 3 or more adjacent counted wells that gave the corresponding titers.

### SDS-PAGE/Western blotting.

Cells were washed twice in PBS, lysed in 1× Glasgow lysis buffer (GLB; 1% [vol/vol] Triton X-100, 120 mM KCl, 30 mM NaCl, 5 mM MgCl_2_, 10% [vol/vol] glycerol, 10 mM PIPES [piperazine-*N*,*N*′-bis(2-ethanesulfonic acid)]-NaOH, pH 7.2, with protease and phosphatase inhibitors), and clarified by centrifugation at 2,800 × *g* for 5 min at 4°C, before determining and normalizing the protein concentration by a bicinchoninic acid assay (Pierce). Following separation by SDS-PAGE, proteins were transferred to a polyvinylidene difluoride (PVDF) membrane and blocked in 50% (vol/vol) Odyssey blocking (OB) buffer (LI-COR) in PBS. The membrane was incubated with primary antibodies overnight at 4°C, followed by incubation with secondary antibodies for 2 h at room temperature (RT); both primary and secondary antibodies were prepared in 50% OB buffer. The primary antibodies used were anti-NS5A (sheep, 1:5,000) (21), anti-NS3 (sheep, 1:2,000; prepared in-house), and anti-glyceraldehyde-3-phosphate dehydrogenase (anti-GAPDH; mouse, 1:20,000; Sigma). Secondary antibodies were anti-goat (800 nm) or anti-mouse (700 nm), which were used at 1:5,000, prior to imaging fluorescence using a LI-COR Odyssey Sa infrared imaging system. Quantification of fluorescently labeled Western blots was carried out using Image Studio (v3.1) software (LI-COR) and a background subtraction method.

### Immunofluorescence and confocal microscopy.

Cells were washed once in PBS before fixation for 20 min in 4% (wt/vol) paraformaldehyde (PFA). The cells were subsequently permeabilized in 0.2% (vol/vol) Triton X-100–PBS before immunostaining with the antibody denoted below. The primary antibodies used were anti-NS5A (sheep, 1:1,000), anti-NS3 (sheep, 1:300; prepared in-house), anti-dsRNA (mouse, 1:200; Scicons), and anti-PI4P (mouse, 1:100; Echelon). Various fluorescently conjugated secondary antibodies were used at 1:1,000 (Life Technology). Note that when conducting immunostaining for dsRNA, all reagents were prepared to be RNase free by DEPC treatment. Lipid droplets were stained using the BODIPY(558/568)-C12 dye at 1:1,000 (Life Technology), which was added at the same time as the fluorescent secondary antibody. Confocal microscopy images were acquired on a Carl Zeiss LSM 700 inverted microscope, and postacquisition analysis was conducted using either Zeiss Zen 2012 or Fiji (v1.49) software (22).

### In-cell labeling of the SNAP/CLIP tag.

Huh7 cells harboring SNAP/CLIP replicons were fixed in 4% PFA and permeabilized in 0.2% Triton X-100–PBS to which 0.9 mM CaCl_2_ and 0.5 mM MgCl_2_ were added (PBS++). The SNAP and CLIP substrates were dissolved to 1 mM in dimethyl sulfoxide, before a 1 μM SNAP/CLIP labeling solution was prepared in PBS++ containing 10% (vol/vol) FBS and added to cells for 2 h at RT. For immunostaining of NS5A, antibody was added to SNAP/CLIP labeling solution, followed by secondary antibody staining, as detailed above.

### Quantification of lipid droplets.

For quantification of the LD spatial arrangement, images were acquired by use of the same acquisition parameters described above, but a variable gain was used to ensure the correct exposure. The spatial coordinates of the LDs were determined using the FindFoci function of the GDSC plug-in for Fiji software, with the nuclear envelope being manually outlined (utilizing DAPI [4′,6-diamidino-2-phenylindole] staining as a reference), and the coordinates were generated by Fiji. The distance from each lipid droplet to the nuclear envelope was then determined using trigonometry. LD spatial distribution data were generated for 12 randomly selected cells for each JFH-1 virus variant, and data were combined into a box-and-whisker plot (see Fig. 3E).

### Statistics.

Data sets were analyzed using Student's *t* test, assuming a two-tailed, unequal variance to determine statistically significant differences from the results for the wild type (WT) (*n* = 3 or greater throughout).

## RESULTS

### Phosphorylation of serine 225 is important for genome replication but not assembly of infectious virus.

**FIG 1:**
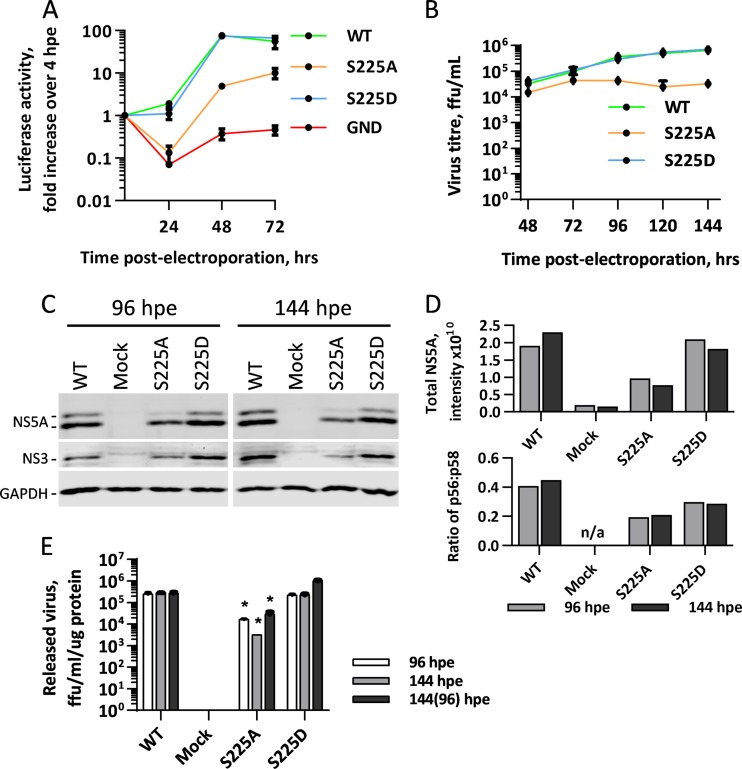
Characterization of the serine 225 phosphorylation site. (A) Huh7 cells were electroporated with *in vitro* transcripts of the indicated mSGR-luc-JFH-1 genomes, seeded into plates with a 96-well format, and incubated for 4, 24, 48, and 72 hpe. Luciferase activity was determined at 24, 48, and 72 hpe and normalized to the value at 4 hpe. (B) Huh7 cells were electroporated with *in vitro* transcripts of the indicated mJFH-1 virus genomes and seeded into plates with a 6-well format, cell supernatant was harvested at the denoted time points, and the titer of the released virus was subsequently determined by a focus-forming assay. (C) At 96 and 144 hpe, cells from the assay whose results are presented in panel B were lysed and analyzed by SDS-PAGE/Western blotting. (D) Total NS5A levels and the ratio of hyperphosphorylation to basal phosphorylation were quantified from fluorescent Western blots. (E) In order to account for the differences in cell growth rate, the titer of the released virus was normalized to the total protein concentration, and cells were also passaged 1:4 at 96 hpe before harvesting the supernatants of the released virus at 144 hpe. ffu, focus-forming units.

Previously, we identified serine 225 to be one of a number of serines within the LCS I of NS5A that is phosphorylated during the normal course of infection. To investigate the role of serine 225 phosphorylation in the virus life cycle, we generated either a phosphoablatant alanine substitution or a phosphomimetic aspartic acid substitution at this residue in the context of both the mSGR-luc-JFH-1 and mJFH-1 constructs by site-directed mutagenesis, as described previously (16). Huh7 cells were then electroporated with *in vitro* transcripts of mSGR-luc-JFH-1, and genome replication was followed over 72 h by determination of luciferase activity. As seen previously (16), the phosphoablatant mutation S225A resulted in a 10-fold reduction in genome replication efficiency, while the phosphomimetic mutation S225D had no discernible effect (Fig. 1A). To investigate whether the phosphorylation of serine 225 had additional effects on the release of infectious virus, *in vitro* transcripts of the mJFH-1 virus with the S225A/D mutations were electroporated into Huh7 cells, and the titer of the released virus was followed over 144 h by a focus-forming assay. Figure 1B shows that the S225A mutant virus had about a 10-fold reduction in infectious virus compared to the WT, while the S225D mutant virus had titers indistinguishable from those of the wild type. This reduction in the titer of released S225A mutant virus correlated with the impairment of replication, in line with the findings of previous studies showing that genome replication is the rate-limiting step in the release of infectious virus (23). These data illustrate that, as previously concluded (16), phosphorylation of serine 225 is important for viral genome replication but not required for the release of infectious virus.

Cell lysates from infected cells were collected at 96 and 144 h postelectroporation (hpe), normalized for protein concentration, and analyzed by SDS-PAGE/Western blotting. It was found that there was a reduction in the level of cellular NS5A to a degree comparable to the reduction in the level of virus replication (Fig. 1C); quantification of the Western blot confirmed this (Fig. 1D). However, when the ratio of hyperphosphorylation to basal phosphorylation was determined, it was found that the S225A mutant exhibited a reduction in the extent of hyperphosphorylation at both early and late time points (Fig. 1D). This correlated with the involvement of the LCS I in hyperphosphorylation previously reported by us and others (14–16).

It was noted that when the protein concentration was normalized for SDS-PAGE, cells infected with the S225A mutant virus were growing at a higher rate than wild-type virus- and S225D mutant virus-infected cells (data not shown). To account for this, the titer of the released virus at 96 and 144 hpe was normalized to the cellular protein concentration, used here as a surrogate for cell number, and it was observed that the S225A mutant still exhibited an approximately 10-fold reduction in the amount of virus released compared to that for the wild type (Fig. 1E). To ensure that cell confluence (known to negatively affect HCV replication) was not affecting the titers of the released virus, electroporated cells were also passaged at 96 hpe, and at 144 hpe, the titer of the released virus was determined and normalized to the cellular protein concentration (Fig. 1E). As noted above, the S225A mutant exhibited a 10-fold reduction in the amount of virus released compared with that for the wild type.

### Ablation of serine 225 phosphorylation alters the subcellular distribution of NS5A.

**FIG 2:**
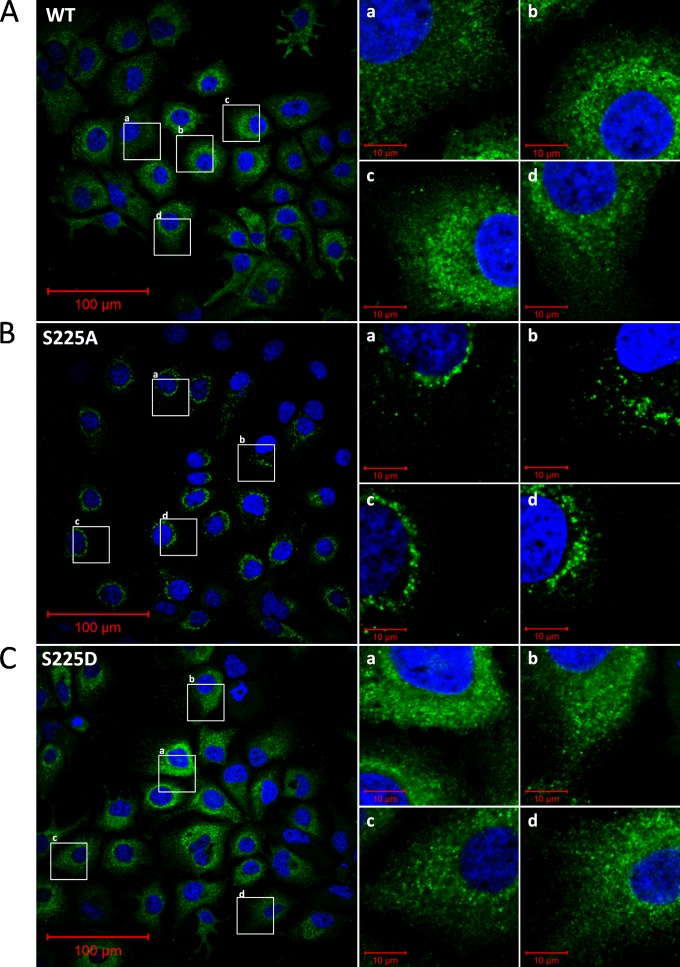
Subcellular distribution of wild-type NS5A and NS5A with mutations at serine 225 in JFH-1-infected cells. Huh7 cells were electroporated with *in vitro* transcripts of either wild-type mJFH-1 (A) or the NS5A(S225A) (B) or NS5A(S225D) (C) mutant, and the cells were seeded onto coverslips and incubated for 96 h prior to fixation, immunostaining for NS5A (sheep, 1:1,000), and imaging by confocal microscopy. Panels a to d on the right are higher magnifications of the corresponding boxed areas in the wide-field images.

When virus was titrated by a focus-forming assay, the subcellular distribution of NS5A with the S225A mutation [NS5A(S225A)] was observed to be markedly different from that of the wild-type protein. Typically in wild-type JFH-1-infected cells, NS5A was present throughout the cytoplasm in discrete puncta; however, in cells infected with the S225A mutant virus, NS5A localized into defined, condensed structures. To investigate this further, Huh7 cells were electroporated with *in vitro* transcripts of mJFH-1 virus (the wild type and the S225A and S225D mutants), fixed at 96 hpe, and immunostained for NS5A as described above. As described above, wild-type NS5A was distributed into small, discrete puncta that were abundant and relatively uniformly distributed throughout the cytoplasm (Fig. 2A). In contrast NS5A(S225A) exhibited bright, condensed clusters that were less abundant and typically localized around the nucleus (Fig. 2B). Importantly, the virus with the S225D phosphomimetic mutation exhibited a wild-type phenotype (Fig. 2C), strongly suggesting that the phosphorylation of serine 225 is important for the normal subcellular localization of NS5A.

### The S225A mutation results in relocalization of genome replication complexes.

**FIG 3:**
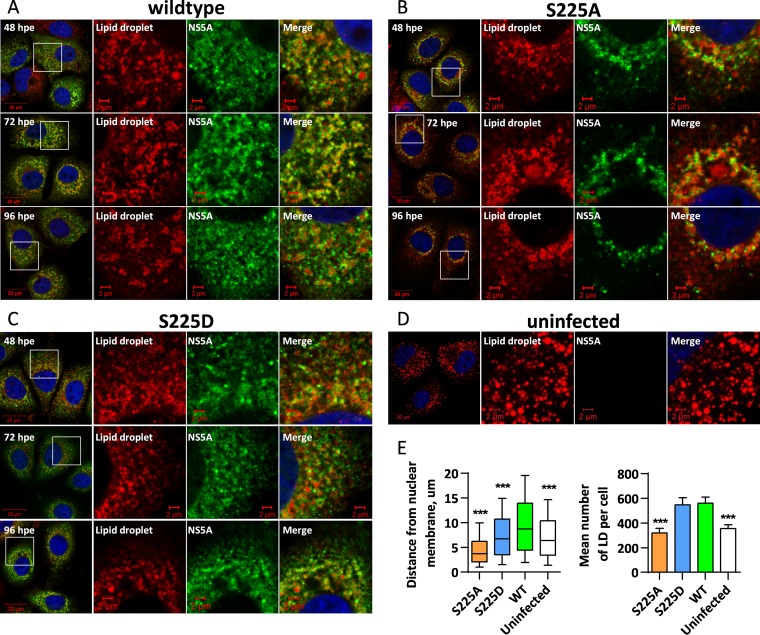
Analysis of lipid droplet redistribution in infected cells. Huh7 cells were electroporated with *in vitro* transcripts of either wild-type mJFH-1 (A) or the NS5A(S225A) (B) or NS5A(S225D) (C) mutant or were not infected (D), and cells were seeded onto coverslips and incubated for 48, 72, and 96 hpe prior to fixation, immunostaining for NS5A and LDs, and imaging by confocal microscopy. Uninfected control cells were also processed at 96 h postseeding. Spatial data for LDs were determined from 12 cells for each virus using the GDSC plug-in for Fiji. (E) These data were used to determine the number of LDs per cell as well as the distance of each LD from the nuclear envelope. Box-and-whisker plots show the 10 to 90% distribution. ***, significant difference (*P* < 0.05) from the results for the wild type.

We then asked whether the relocalization of NS5A(S225A) resulted in any changes to the distribution of other viral and cellular markers of replication complexes. First, we looked at the distribution of lipid droplets (LDs), which serve as a neutral lipid reservoir for the host and have previously been shown to be an essential host factor involved in HCV replication, with both the NS5A and core proteins localizing to their surface in infected cells (9). In the case of both the wild-type virus and the two mutant JFH-1 viruses, NS5A colocalized to the periphery of LDs (Fig. 3A to C), and the distribution of the LDs correlated with that of NS5A. Specifically, in cells infected with the S225A mutant virus, LDs clustered close to the nucleus, whereas in the context of either wild-type or S225D mutant infection, the LDs were more evenly distributed throughout the cytoplasm. In uninfected cells, the distribution of LDs more closely resembled that in wild-type- or S225D mutant-infected cells (Fig. 3D).

To quantify this LD redistribution, we acquired images of 12 randomly selected cells for the wild type and each virus mutant and quantified both the number of LDs and their distance from the nuclear membrane. With this quantitative approach, we saw that in comparison to uninfected cells, wild-type virus-infected cells exhibited a broader distribution throughout the cytoplasm, whereas in the context of the S225A mutation, there was a significant shift of LDs toward the nucleus (Fig. 3E). Interestingly, however, cells infected with virus with the S225D mutation had an intermediate phenotype that resembled that of uninfected cells, where there was a slight shift of LDs toward the nucleus, indicating that this mutation was not quite fully able to recapitulate the effect of phosphorylation at serine 225, at least with respect to the LD distribution (Fig. 3E). With these data, we were also able to interrogate the total number of LDs per cell. There was about a 2-fold increase in the number of LDs in cells infected with either wild-type virus or the S225D mutant viruses compared to the number in naive cells (Fig. 3E). This was not the case for the S225A mutant, where the number of LDs per cell for S225A mutant-infected cells was broadly comparable to that for uninfected cells (Fig. 3E), suggesting that NS5A(S225A) is unable to increase the formation of LDs upon infection.

**FIG 4:**
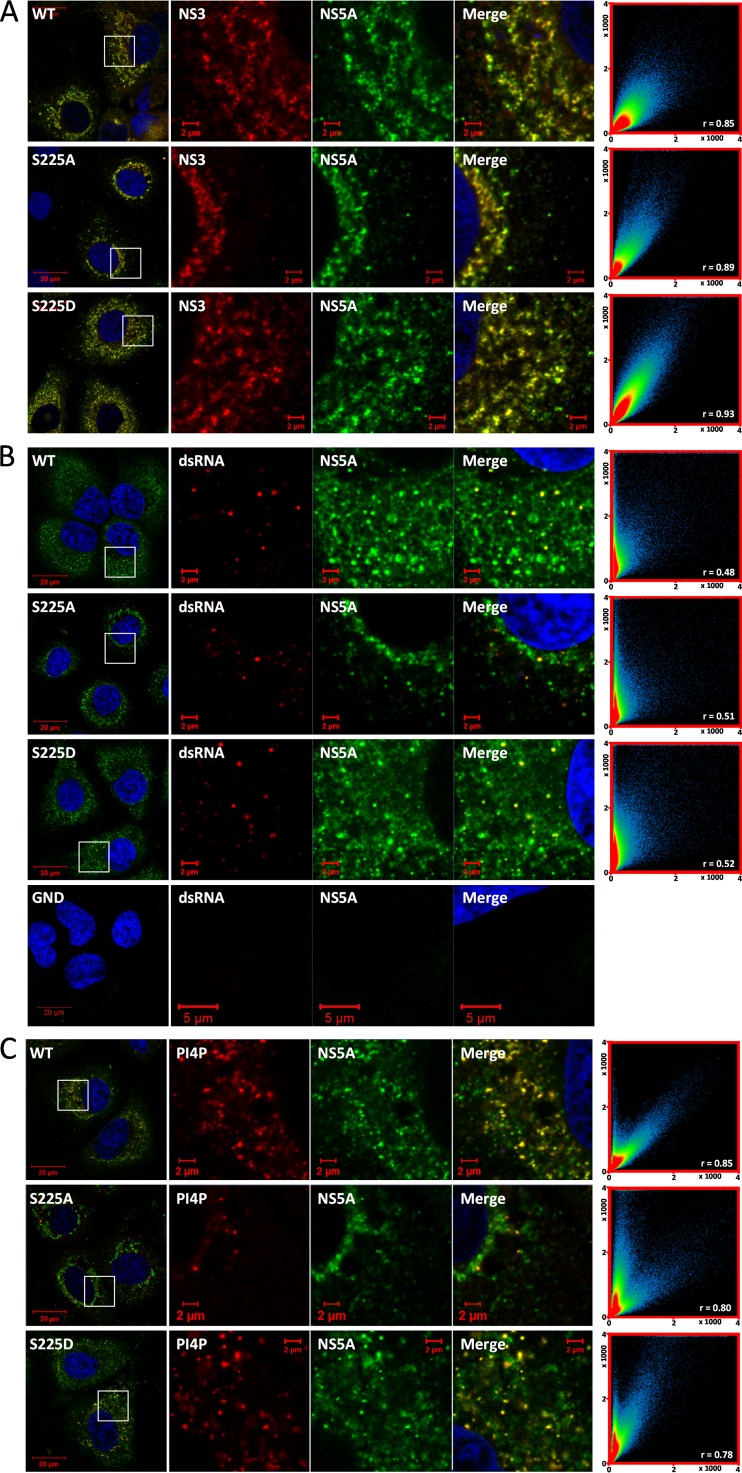
Colocalization of NS5A phosphorylation mutants with host and viral factors. Huh7 cells were electroporated with *in vitro* transcripts of either wild-type mJFH-1 or the NS5A(S225A) or NS5A(S225D) mutant and seeded onto coverslips. At 96 hpe, cells were fixed and immunostained for NS5A and either NS3 (A), dsRNA (B), or the phospholipid PI4P (C). An NS5B GND (nonreplicating) control was included for the dsRNA staining. The colocalization scatter plots on the right are of an area of 200 by 200 μm containing >10 cells and show the correlation of absolute values (*x* and *y* axes, pixel numbers). The correlation value (*r*) is shown for each image.

The viral protease/helicase NS3 is another key component of genome replication complexes; therefore, to further investigate the potential redistribution of these complexes caused by the S225A mutation, we costained infected cells with antibodies to both NS5A and NS3. As expected, NS3 extensively colocalized with NS5A in all cases, such that in the context of NS5A(S225A) it exhibited a restricted perinuclear distribution, whereas for both wild-type NS5A and NS5A with the S225D mutation [NS5A(S225D)], NS3 was extensively distributed throughout the cytoplasm (Fig. 4A). A similar phenotype was observed for the distribution of double-stranded RNA (dsRNA), a hallmark of viral genome replication that can be detected using a specific antibody, J2 (24). As shown in Fig. 4B, in cells infected with mJFH-1, the dsRNA antibody detected small, bright puncta, typically of a uniform size and with a distribution throughout the cytoplasm. In cells electroporated with a replication-defective mutant (GND), however, there was little to no reactivity toward the dsRNA antibody, suggesting that this staining was specific to cells undergoing virus genome replication (Fig. 4B). Furthermore, this dsRNA staining could be observed at very early time points postinfection (24 h), further supporting the notion that these puncta are active replication complexes and are not the result of the accumulation of structured RNA in stress granules (data not shown). While the distribution of dsRNA-positive puncta was not as extensive as that of lipid droplets (Fig. 3) or NS3 (Fig. 4A), it again correlated with the distribution of NS5A. Thus, in cells infected with either wild-type or S225D mutant viruses, dsRNA-positive puncta could be seen throughout the cytoplasm, whereas in cells infected with the S225A mutant virus, they were less numerous and restricted to a perinuclear distribution. The limited distribution of the dsRNA-positive puncta may be a consequence of the lack of sensitivity of the antibody or may indicate that there are only a limited number of active replication complexes and that the number of active replication complexes is much less than the total number of structures containing NS3 and NS5A.

Lastly, we investigated the distribution of a host cell factor, the phospholipid phosphatidylinositol 4-phosphate (PI4P). It has been well established that infection by HCV and many other RNA viruses results in the increased production of PI4P lipids, and it is widely accepted that this is one of the mechanisms through which HCV is able to induce the formation of the membranous web (5–7). Using an antibody specific to PI4P, we showed that in cells infected with wild-type or S225D mutant viruses, PI4P levels were elevated in comparison to those in uninfected cells (data not shown) and that there was a strong colocalization between NS5A and PI4P, with PI4P typically forming small irregular puncta (Fig. 4C). In contrast, cells infected with the S225A mutant virus showed a dramatic reduction in the abundance of PI4P puncta and their relocalization to a perinuclear distribution along with NS5A (Fig. 4C).

From the findings of this confocal microscopy and immunofluorescence analysis, we conclude that the phosphoablatant mutation S225A in NS5A is sufficient to induce the relocalization of numerous factors associated with HCV genome replication. It is important to note that we did not observe a loss of colocalization of NS5A with these factors, reflecting the fact that NS5A(S225A) retained the ability to replicate and release infectious virus.

### The relocalization of NS5A(S225A) and viral/host factors is not a result of reduced HCV replication.

It was important to establish that the phenotype of reduced HCV replication was specifically the result of the loss of phosphorylation at serine 225 and not the result of the lower levels of replication (and, so, viral proteins) that are observed with virus containing NS5A with the S225A mutation. To achieve this, we utilized two previously characterized mutations within domain II of NS5A, P315A and L321A, that had previously been shown to impair (approximately 10-fold) virus replication similarly to S225A (20).

**FIG 5:**
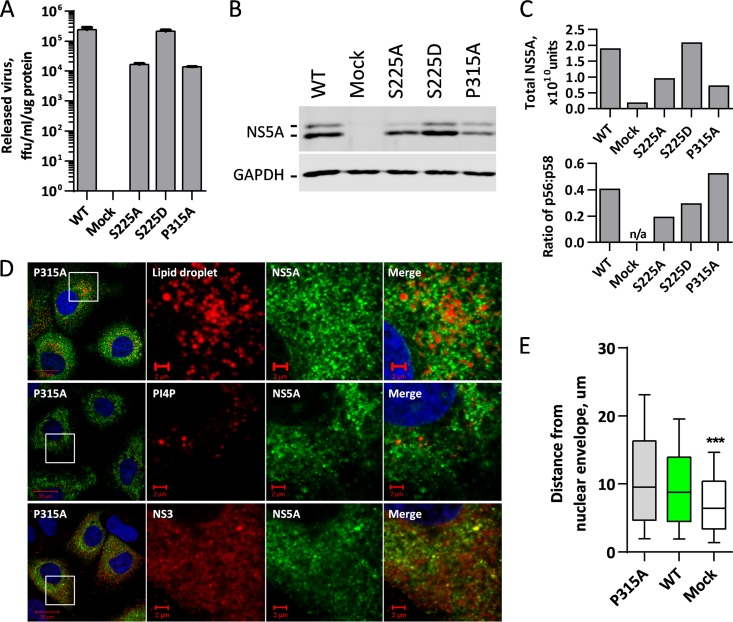
An NS5A domain II mutation that impairs replication does not have the same redistribution phenotype as NS5A(S225A). Huh7 cells were electroporated with *in vitro* transcripts of either wild-type mJFH-1 or the NS5A(S225A), NS5A(S225D), or NS5A(P315A) mutant. (A) The titer of the released virus was determined at 96 hpe by a focus-forming assay. The virus titer was normalized to the cellular protein concentration in order to account for differences in cell growth rates. (B) At 96 hpe, cells were lysed and analyzed by SDS-PAGE/Western blotting for NS5A and GAPDH. (C) Total NS5A levels and the ratio of hyperphosphorylation to basal phosphorylation were quantified from fluorescent Western blots. (D) At 96 hpe, electroporated cells were fixed and immunostained for NS5A and either LDs, PI4P, or NS3, as described in the legend to Fig. 4. (E) Spatial data for LDs were determined from 12 cells for each virus using the GDSC plug-in for Fiji. Box-and-whisker plots show the 10 to 90% distribution. ***, significant difference (*P* < 0.05) from the results for the wild type.

As expected, an approximately 10-fold reduction in the amount of virus that was released was exhibited for both the mutant virus containing NS5A with the P315A mutation [NS5A(P315A)] (Fig. 5A) and the mutant virus containing NS5A with the L321A mutation [NS5A(L321A)] (data not shown); this amount was comparable to that achieved with virus with the S225A mutation, confirming that the S225A, P315A, and L321A mutations impair virus replication to similar levels. Both mutant viruses exhibited a reduction in the levels of NS5A expression similar to that seen for virus with the S225A mutation (Fig. 5B and C and data not shown) and exhibited a ratio of NS5A hyperphosphorylation to basal phosphorylation similar to that for the wild type (Fig. 5C and data not shown) but different from that for both S225 mutants, consistent with the previously proposed notion that S225 is a site of phosphorylation in the hyperphosphorylated species of NS5A (16). Therefore, if the phenotype of NS5A relocalization was the result of reduced replication, it would be expected that both the P315A and L321A mutants would show the same phenotype.

Huh7 cells infected with mutant viruses with the NS5A(P315A) (Fig. 5D) or NS5A(L321A) (data not shown) mutation were analyzed by confocal microscopy for the abundance and distribution of NS5A and LDs. Reassuringly, the distribution of NS5A in cells infected with these mutants was indistinguishable from that in cells infected with the wild type, with no evidence of the restriction to a perinuclear distribution. Quantitative analysis revealed no significant difference between the wild type and the two mutants with regard to the distance of the LDs from the nuclear membrane (Fig. 5E). Similar results were seen for the distribution of NS3 and PI4P (Fig. 5D), as well as for that of dsRNA (data not shown). It is noteworthy that although the distribution of PI4P lipids in NS5A(P315A) virus-infected cells was similar to that in wild type-infected cells, there was an obvious reduction in their quantity, suggesting that this mutation might have an independent effect on NS5A-mediated PI4K activation. This observation notwithstanding, we conclude that the effect of the S225A mutation on the distribution of replication complex components cannot be explained by a reduction in replicative fitness; rather, it is specifically the loss of phosphorylation at serine 225 that is responsible for this phenotype.

### The S225A mutant phenotype does not correlate with a loss of hyperphosphorylation.

**FIG 6:**
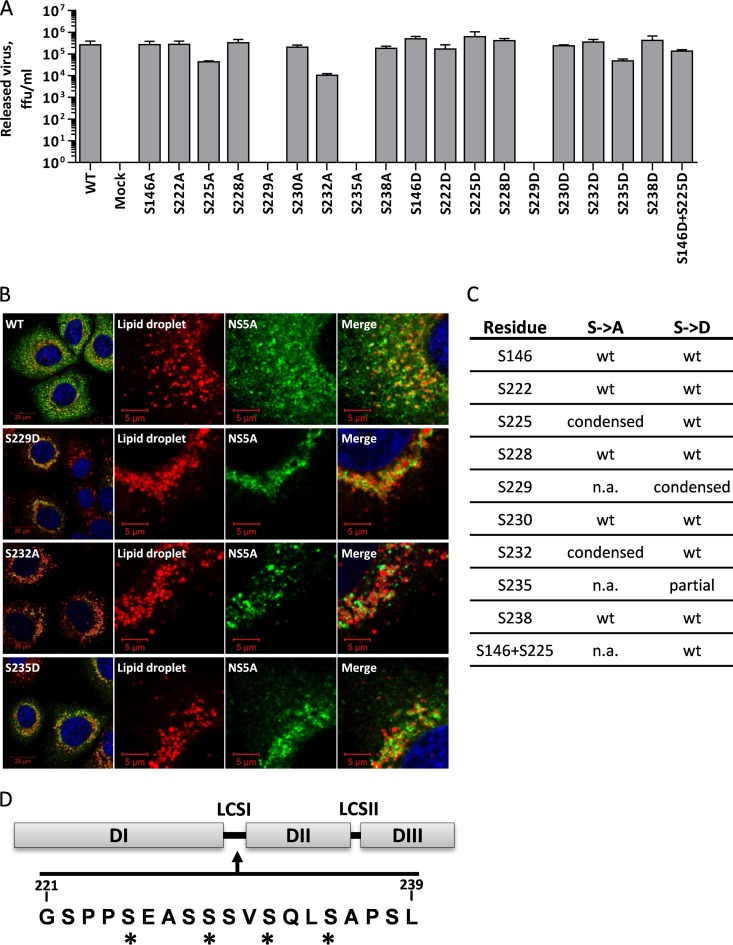
Mutants with other serine mutations in LCS I share the condensed redistribution phenotype with the S225A mutant. (A) Huh7 cells were electroporated with *in vitro* transcripts of either wild-type mJFH-1 or the indicated mutants. The titer of released virus was determined at 96 hpe by a focus-forming assay. (B) At 96 hpe, electroporated cells were fixed and immunostained for NS5A and LDs, as described in the legend to Fig. 3. Only the results for the three mutants that shared the phenotype are shown here for comparison with the wild type. See Fig. S1 in the supplemental material for the complete data set. (C) Summary table of mutant phenotypes. n.a., not available (did not replicate). (D) Amino acid sequence of LCS I. *, serine residues with a redistribution phenotype; DI, DII, and DIII, domains I, II, and III, respectively.

Although our original observation was made during routine virus titration experiments, we had not screened an entire panel of mutants with serine mutations within LCS I. Given the complexity of the phosphorylation patterns within this region and the potential role of S146 in regulating hyperphosphorylation (16), we therefore generated a complete panel of mutants with either phosphomimetic or phosphoablatant mutations in all 8 serines in LCS I and S146 and examined their phenotypes by both virus titration and immunofluorescence. Consistent with the findings of our previous analysis (16) and those of Masaki et al. (15), most mutations had no effect on virus titer; however, the S225A, S232A, and S235D mutant viruses all exhibited a significant reduction in titer (approximately 10-fold) (Fig. 6A). Cells infected with the S232A and S235D mutants also shared the restricted perinuclear distribution of NS5A and LD (Fig. 6B). As demonstrated previously (15), the S229D mutation did not result in the production of any infectious virus; however, the S229D mutant exhibited limited RNA replication and protein expression (15, 16), and the distribution of both NS5A and LDs in S229D mutant-infected cells was again restricted to a perinuclear region. We conclude that the phosphorylation of different serines within LCS I can have opposing effects on the subcellular distribution of NS5A.

Lastly, we examined the phenotype of a double mutant, the S146D and S225D mutant, that we had previously shown replicated as well as the wild type but in which hyperphosphorylation was almost completely abolished (16). The distribution of NS5A in cells infected with this mutant was comparable to that in cells infected with the wild type (see Fig. S1 in the supplemental material), confirming that the phenotype of a restricted perinuclear distribution for the S225A mutant could not be explained by a defect in hyperphosphorylation. As an aside, none of the mutants (including the S225A mutant) exhibited any significant differences in sensitivity to daclatasvir (DCV) from that of the wild type. The 50% effective concentrations (EC_50_s) ranged from 10 to 57 pM (in our hands, a wild-type JFH-1 replicon had an EC_50_ of 57 pM), and although treatment with DCV reduced the abundance of NS5A, it did not alter the widespread cytoplasmic distribution (data not shown).

### The phenotype of the S225A mutant is not the result of cell culture conditions.

To enable us to interrogate further the phenotype of the S225A mutant, we utilized the SNAP and CLIP tag (NEB) technology. This complementary tag system allows the specific conjugation of either benzylguanine or benzylcytosine derivatives, typically fluorescently labeled, to the SNAP or CLIP tag, respectively. This approach has recently been described for NS5A (25), and we reasoned that it would enable us to differentially label wild-type NS5A and the S225A and S225D mutants within the same population of cells. The advantage of the SNAP and CLIP tags over the traditional green fluorescent protein (GFP) and red fluorescent protein tags is a combination of the flexibility offered with the SNAP/CLIP system and the high sequence similarity between the SNAP and CLIP tags.

**FIG 7:**
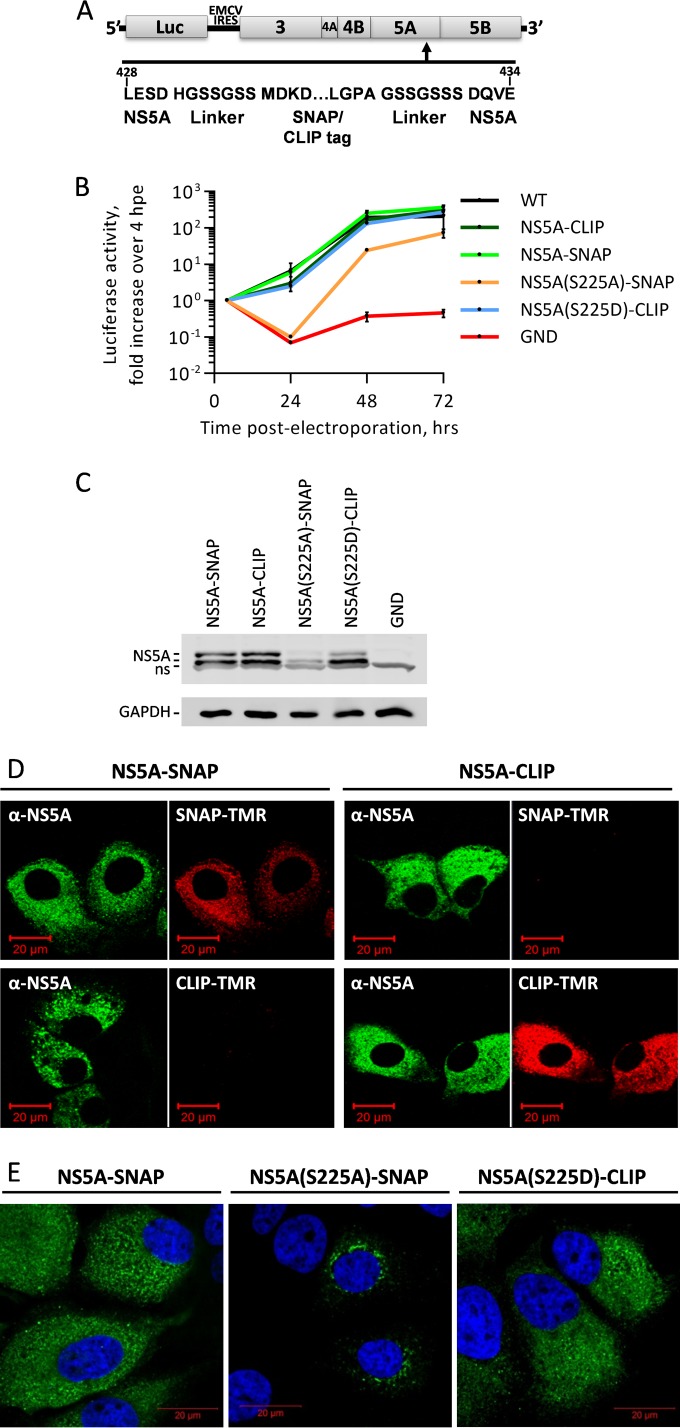
Generation and validation of mSGR-luc-JFH-1 replicons containing either NS5A-SNAP or NS5A-CLIP fusions. (A) Schematic of the mSGR-luc-JFH-1 replicon showing the region of NS5A where the SNAP/CLIP tag was inserted, along with the flanking, flexible linker sequences. The amino acid numbering is for JFH-1 NS5A. (B) Huh7 cells were electroporated with the indicated mSGR-luc-JFH-1 replicon RNAs, and replication was followed over 72 h by determining luciferase activity, which is shown normalized to the activity at 4 hpe. (C) Cells were lysed at 72 hpe and analyzed by SDS-PAGE/Western blotting for NS5A and GAPDH. A nonspecific band (ns) was seen at equal levels in all lysates (D). Huh7 cells electroporated with the NS5A-SNAP (left) or NS5A-CLIP (right) replicon RNAs were also seeded onto coverslips and at 72 hpe were fixed and immunostained for NS5A, as well as labeled with either SNAP-TMR or CLIP-TMR, before imaging by confocal microscopy. (E) Huh7 cells electroporated with the indicated mSGR-luc-JFH-1 replicon RNAs were seeded onto coverslips, fixed at 96 hpe, and immunostained for NS5A.

The SNAP/CLIP tags were initially introduced into the wild-type mSGR-luc-JFH-1 replicon (19) at position 431 in NS5A (domain III), as this site has previously been shown to tolerate large insertions (Fig. 7A). We confirmed that the insertion of either tag had no discernible effect on the replication kinetics of the mSGR-luc-JFH-1 replicon compared to those of the untagged wild-type replicon (Fig. 7B). As the SNAP and CLIP tags share very high sequence similarity yet retain different substrate specificities, it was important to establish that the insertion of the tag at an internal position did not introduce conformation changes that affected the substrate specificity. As such, cells harboring the mSGR-luc-JFH-1-NS5A-SNAP (NS5A-SNAP) replicon were fixed at 72 hpe, immunostained for NS5A, and labeled with a CLIP-tetramethylrhodamine (TMR) fluorophore. The converse, where the mSGR-luc-JFH-1-NS5A-CLIP (NS5A-CLIP) replicon was labeled with the SNAP-TMR fluorophore, was also carried out. Figure 7D shows that both the SNAP and CLIP tags retained both catalytic activity and specificity toward the appropriate SNAP/CLIP substrate, with SNAP-TMR labeling only NS5A-SNAP and CLIP-TMR labeling only NS5A-CLIP. The corresponding immunostaining for NS5A also showed a very high degree of correlation, confirming that there was no subpopulation of either NS5A-SNAP or NS5A-CLIP that was not labeled with the SNAP/CLIP substrates.

We proceeded to use the SNAP/CLIP system to establish whether there was a hitherto unknown environmental factor that was inducing the phenotype of the S225A mutant. To do this, we generated mSGR-luc-JFH-1-NS5A(S225A)-SNAP [NS5A(S225A)-SNAP] and mSGR-luc-JFH-1-NS5A(S225D)-CLIP [NS5A(S225D)-CLIP] replicons. As shown in Fig. 7B, these two mutants retained their replication phenotypes (a 10-fold impairment of replication for the S225A mutant and wild-type replication for the S225D mutant) when expressed as SNAP/CLIP fusions. Analysis of cell lysates by Western blotting showed an abundance and hyperphosphorylation/basal phosphorylation ratios of NS5A comparable to those observed for the untagged mutants (Fig. 7C). As seen in the context of virus-infected cells (Fig. 3 and 4), cells harboring the NS5A(S225A)-SNAP replicon exhibited both a reduced abundance and a restricted perinuclear distribution of NS5A (Fig. 7E) in comparison to those for cells harboring either the wild-type (SNAP tagged) or the NS5A(S225D)-CLIP replicon.

**FIG 8:**
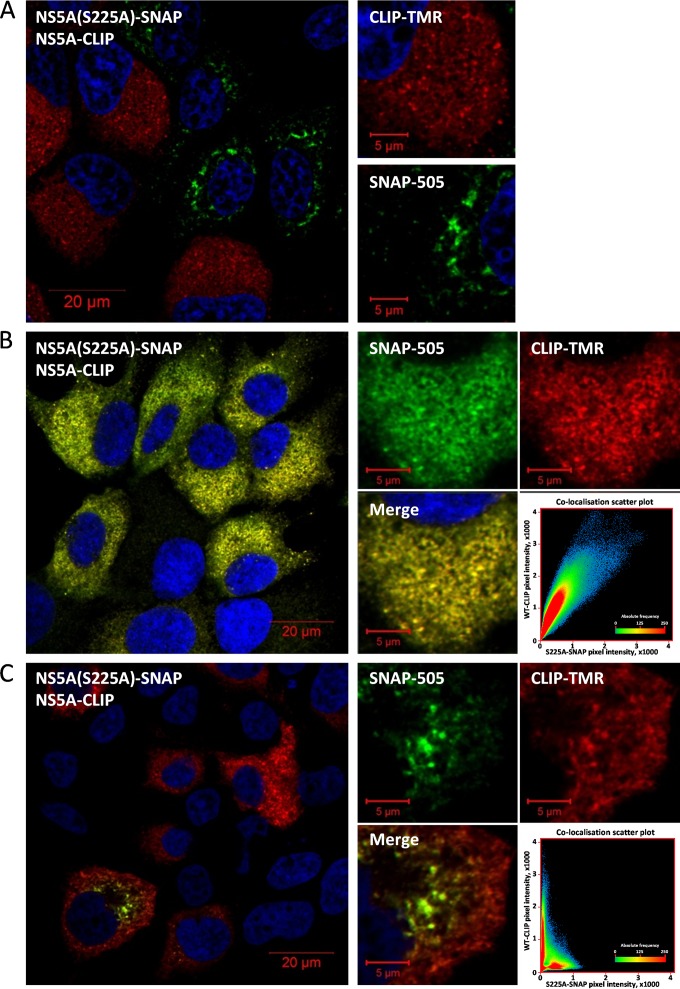
The redistribution of NS5A(S225A) is not the result of an environmental factor and can be partially transcomplemented by wild-type NS5A. (A) Huh7 cells electroporated with either mSGR-luc-JFH-1-NS5A-CLIP or mSGR-luc-JFH-1-NS5A(S225A)-SNAP RNAs were coseeded onto the same coverslip, and at 96 hpe, cells were fixed and labeled with either SNAP-505 (green) or CLIP-TMR (red), before imaging by confocal microscopy. (B, C) *In vitro* transcripts of mSGR-luc-JFH-1-NS5A-CLIP and mSGR-luc-JFH-1-NS5A(S225A)-SNAP were pooled prior to coelectroporation into Huh7 cells and seeding onto coverslips. At 96 hpe, cells were fixed and labeled with SNAP-505 and CLIP-TMR, prior to imaging by confocal microscopy. (B) Example of a population of Huh7 cells showing colocalization between wild-type NS5A(CLIP) and NS5A(S225A)-SNAP. (C) Example of an Huh7 cell showing a difference in cellular localization between NS5A(CLIP) and NS5A(S225A)-SNAP. The colocalization scatter plots at the bottom right of panels B and C are of individual cells and show the correlation of absolute values.

To confirm that the S225A phenotype was not due to an environmental factor, such as differences in cell confluence, we electroporated either mSGR-luc-JFH-1-NS5A-CLIP or mSGR-luc-JFH-1-NS5A(S225A)-SNAP replicon RNAs into Huh7 cells and coseeded the electroporated cells into single cultures. By confocal microscopy, we observed that within the same population of cells, in the same field of view, those harboring the wild-type NS5A-CLIP replicon had a normal distribution of NS5A, while those harboring the NS5A(S225A)-SNAP replicon continued to show the restricted NS5A localization (Fig. 8A).

To further investigate the NS5A(S225A) phenotype, we utilized the SNAP/CLIP tag technology to ask if NS5A(S225A) could be transcomplemented by wild-type NS5A. In our experience, the simultaneous introduction of two replicons into a single cell is a rare event, for reasons that are not clear, particularly given the ratio of RNA molecules to cells in a typical electroporation experiment. However, after coelectroporation of mSGR-luc-JFH-1-NS5A-CLIP and mSGR-luc-JFH-1-NS5A(S225A)-SNAP replicon RNAs into Huh7 cells, we did observe some cells that were stained with both SNAP-505 and CLIP-TMR fluorophores, consistent with the presence of both actively replicating replicons in a single cell. In these cells, we observed two distinct phenotypes: either the presence of wild-type NS5A-CLIP was sufficient to restore the localization of NS5A(S225A)-SNAP to that of the wild type, resulting in a broad distribution of both and high levels of colocalization (Fig. 8B), or the distribution of NS5A(S225A)-SNAP remained in bright, condensed, and amorphous structures, with wild-type NS5A-CLIP being distributed normally throughout the same cell (Fig. 8C).

## DISCUSSION

We present here the results of our investigation of the phenotype of mutants with mutations at residue serine 225 in NS5A of JFH-1. We propose that the phosphorylation of serine 225 is important for genome replication; mutation of this residue to alanine (S225A) resulted in a relocalization of NS5A and other components of the HCV genome replication complex (NS3, dsRNA, and PI4P lipids) to a perinuclear distribution, in comparison to either wild-type or a phosphomimetic (aspartic acid) substitution (S225D). This altered subcellular localization was accompanied by a 10-fold reduction in genome replication compared with the level of replication of wild-type or S225D mutant virus. This phenotype, together with the reduction in genome replication, was shared with mutants with three other mutations in LCS I (S229D, S232A, and S235D) but, importantly, was not seen for other NS5A mutants with a similar replication defect (P315A and L321A mutations). These data are consistent with serine phosphorylation in LCS I either positively (phosphorylation at S225 and S232) or negatively (phosphorylation at S229 and S235) regulating the formation and distribution of replication complexes. This conclusion also fits well with the concept of a phosphorylation-mediated switch of NS5A functions between replication and virus assembly, although the mechanics of such a switch remain elusive. Intriguingly, S232 corresponds to the residue which was the target for a dramatic culture adaptation (20,000-fold) in the context of genotype 1b replicons (S2204I in polyprotein numbering). Despite the conservation of the sequence of LCS I between HCV genotypes, these observations suggest that this does not manifest in a concomitant conservation of function, as neither we (Fig. 6A) nor others (15) have seen any major effect of mutations at this residue on JFH-1 virus replication. In the future, it will be important to compare the sites of phosphorylation and phenotypes in HCV isolates with a range of genotypes to address these questions.

How might the altered distribution of the mutant NS5A be explained? A clue may come from the experiments examining the distribution of PI4P lipids (Fig. 4C). PI4P lipids are produced by PI4K, and previous work from other laboratories (5–7) has demonstrated that the PI4KIIIα isoform is required for HCV genome replication. Indeed, PI4KIIIα has been shown to bind to NS5A, with the binding site mapping to a short region at the C terminus of domain I (residues 202 to 210) (26), and disruption of this interaction had a profound effect on replication (100-fold reduction), blocked the stimulation of PI4P formation, and led to a collapse of the membranous web. In this regard, the restricted distribution and clustering of NS5A seen in the context of the S225A mutation are reminiscent of the NS5A localization seen in cells silenced for expression of PI4KIIIα (7). We therefore propose that the replication defect of S225A may be explained in part by the inability of this NS5A mutant protein to effectively recruit and activate PI4KIIIα. This simplistic explanation is complicated by the results of experiments in which we expressed NS5A in the context of an NS3-5B polyprotein from a cytomegalovirus promoter-driven plasmid vector (data not shown). In this situation, the nonstructural proteins can form a replication complex, but in the absence of a cognate RNA template, no genome replication can occur. Intriguingly, under these conditions the S225A phenotype is not apparent: the distribution and abundance of both NS5A and PI4P in transfected cells are very similar for both the wild type and the S225A mutant. Thus, we would further propose that the function of S225 phosphorylation in regulating interactions between NS5A and PI4KIIIα (and/or other cellular components) is manifest only in the context of a replication complex that is actively replicating the viral genome. What is difficult to reconcile is the observation that inhibiting the interaction between NS5A and PI4KIIIα increased the hyperphosphorylation of NS5A (26), concomitant with a reduction in virus genome replication. In contrast, our data show a loss of hyperphosphorylation of S225A (Fig. 1D), and indeed, our previous data (16) failed to indicate a correlation between hyperphosphorylation and replication. This discrepancy serves to highlight the complexity of NS5A phosphorylation, particularly regarding the multiple phosphorylation events within LCS I. There is a need to fully characterize both the kinases involved and the consequences of phosphorylation to clarify the function of these posttranslational modifications in the virus life cycle.

An alternative explanation for the restricted distribution of the S225A mutant NS5A is that it was impaired in its ability to traffic around the cytoplasm. In an elegant set of experiments using both tetracysteine and GFP-tagged NS5A-containing viruses, it was recently shown that NS5A was present in two classes of motile structures. The majority were relatively static, but some exhibited rapid, long-range, and sporadic movement throughout the cytoplasm (25). The rapid movement was shown to involve the microtubule network and was dynein dependent. It is thus formally possible that NS5A(S225A) is impaired in interactions with microtubules or dynein, preventing its movement around the cytoplasm and leading to its accumulation in the perinuclear region. However, we do not believe that this is the case because live cell imaging of either wild-type or S225A/D mutant viruses in which NS5A was Emerald-GFP (emGFP) tagged did not reveal any gross differences in the motility of NS5A-positive structures (see Videos S1 to S3 in the supplemental material). In addition, a defect in dynein-dependent trafficking might intuitively be expected to result in a distribution around the periphery of the cell, as dynein moves toward the minus end of microtubules, which is usually at the cell center.

The use of the SNAP/CLIP tagging system allowed us to differentially label NS5A(S225A)-SNAP and wild-type NS5A-CLIP within one population of cells, thereby visualizing in the same field of view cells that were harboring either S225A or wild-type replicons. Importantly, this analysis demonstrated that the distribution of NS5A(S225A) was still markedly different from that of the wild-type protein (Fig. 7E), illustrating that the altered localization of NS5A was not due to an environmental factor. However, the SNAP/CLIP system also revealed that the presence of wild-type NS5A was able to complement the S225A defect in only a proportion of cells. This suggests that additional, as yet undefined, stochastic factors, such as the stage of the cell cycle or the availability of cellular factors, may contribute to the observed phenotype.

In conclusion, this study highlights an important role of phosphorylation in regulating the function of NS5A. Clearly, there is much to do to define the mechanisms underpinning this effect; in particular, we need to understand the molecular interactions that are modulated by the phosphorylation of NS5A at serine 225 and other serines in LCS I. While a number of candidates, such as PI4KIIIα, are already apparent, there are many other NS5A-interacting proteins that should be considered, and a proteomics approach to compare the interactome of both the wild-type and mutant forms of NS5A is an appropriate way forward. Such experiments are under way in our laboratory.

## Supplementary Material

Supplemental material
