# Supplementary material for: Serine Phosphorylation of the Hepatitis C Virus NS5A Protein Controls the Establishment of Replication Complexes
Source: J Virol. 2014 Dec 31;89(6):3123–35. doi: 10.1128/JVI.02995-14 (PMC4337517; doi:10.1128/JVI.02995-14)
Supplement: Supplemental material [file supp_89_6_3123__index.html]

Serine Phosphorylation of the Hepatitis C Virus NS5A Protein Controls the Establishment of Replication Complexes — Supplemental material 

# Serine Phosphorylation of the Hepatitis C Virus NS5A Protein Controls the Establishment of Replication Complexes

## Supplemental material

**Files in this Data Supplement:**

- Supplemental file 4 -

  Fig. S1 (Subcellular distribution of wild-type and serine mutant NS5A in JFH-1-infected cells.)

  Fig. S2 (Wide-field images of Huh7 cells infected with JcINS5A-emGFP viruses used for subsequent video capture.)

  PDF, 2.4M
- Supplemental file 1 -

  Video S1 (Wild-type NS5A.)

  AVI, 18M
- Supplemental file 2 -

  Video S2 (S225A mutant NS5A.)

  AVI, 14M
- Supplemental file 3 -

  Video S3 (S225D mutant NS5A.)

  AVI, 19M
