# Supplementary material for: Serine Phosphorylation of the Hepatitis C Virus NS5A Protein Controls the Establishment of Replication Complexes
Source: J Virol. 2014 Dec 31;89(6):3123–35. doi: 10.1128/JVI.02995-14 (PMC4337517; doi:10.1128/JVI.02995-14)
Supplement: Supplemental material [file JVI.02995-14_zjv999090147so4.pdf]

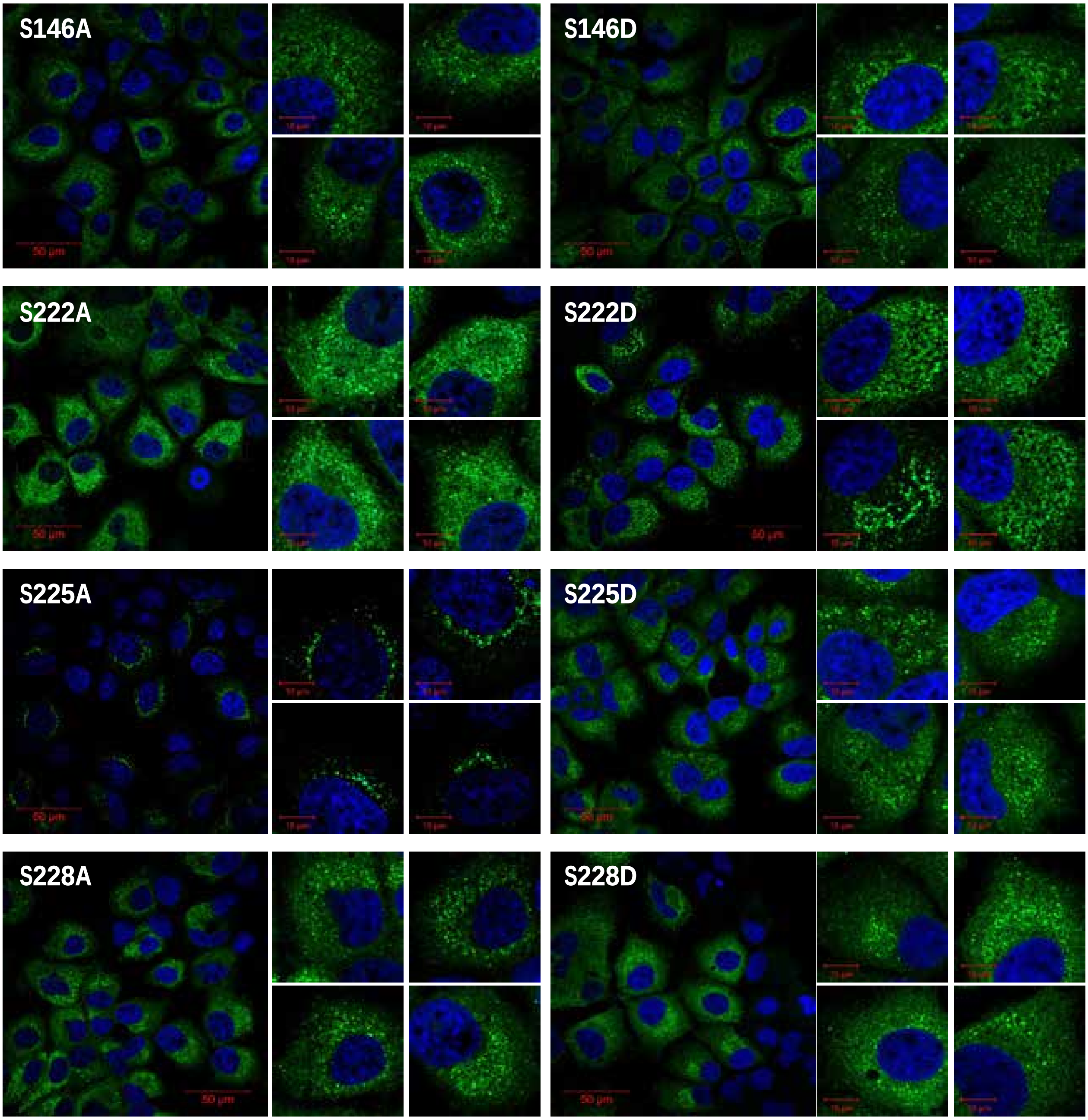


**Supplementary Figure 1a. Sub-cellular distribution of wildtype and serine mutant NS5A in JFH-1 infected cells.** Huh7 cells were electroporated with *in vitro* transcripts of the indicated NS5A mutant JFH-1 viruses. Cells were seeded onto coverslips and incubated for 96 h prior to fixation, immunostaining for NS5A (sheep, 1:1000) and imaging by confocal microscopy.

.

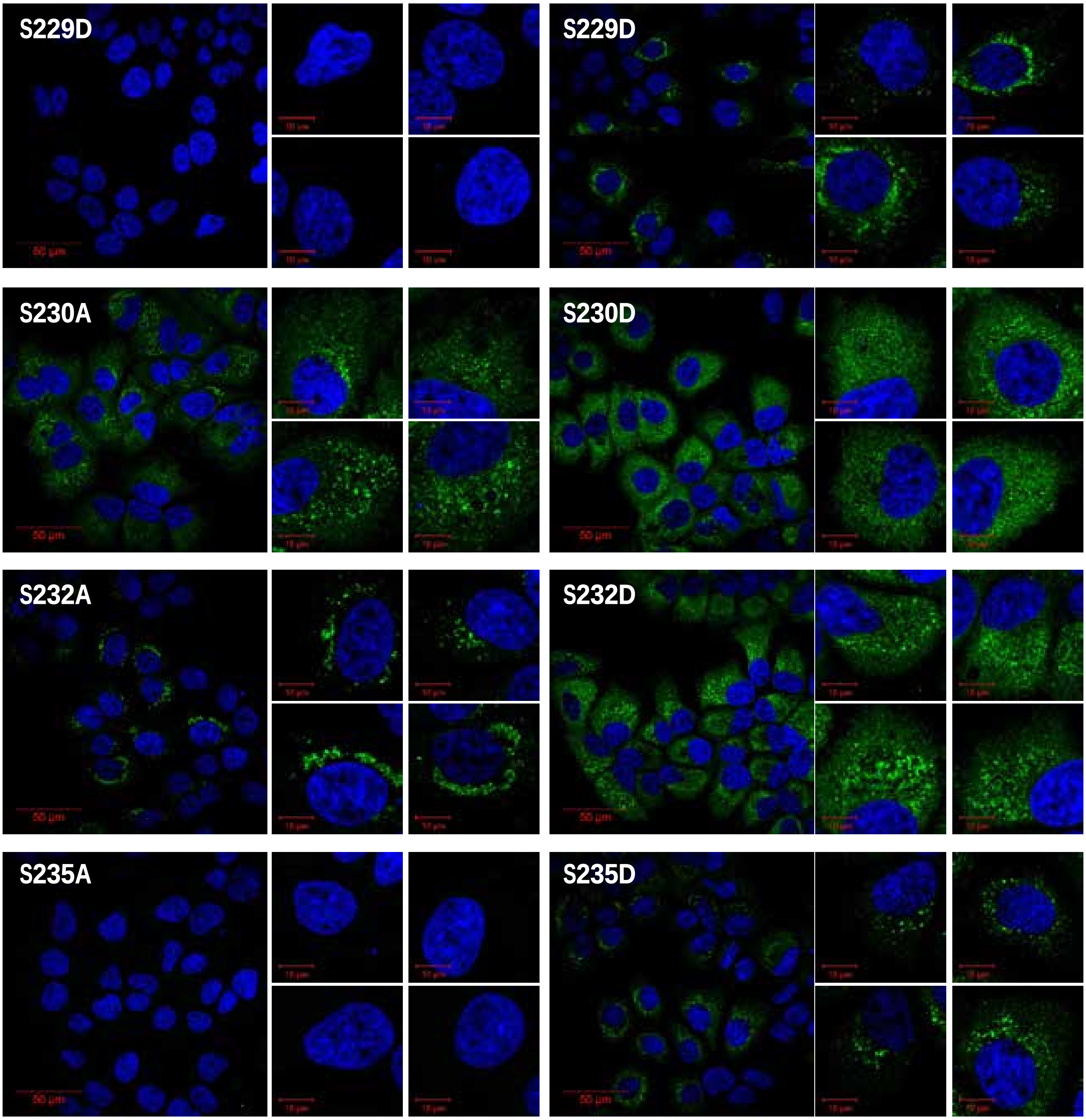


**Supplementary Figure 1b. Sub-cellular distribution of wildtype and serine mutant NS5A in JFH-1 infected cells.** Huh7 cells were electroporated with *in vitro* transcripts of the indicated NS5A mutant JFH-1 viruses. Cells were seeded onto coverslips and incubated for 96 h prior to fixation, immunostaining for NS5A (sheep, 1:1000) and imaging by confocal microscopy.

.

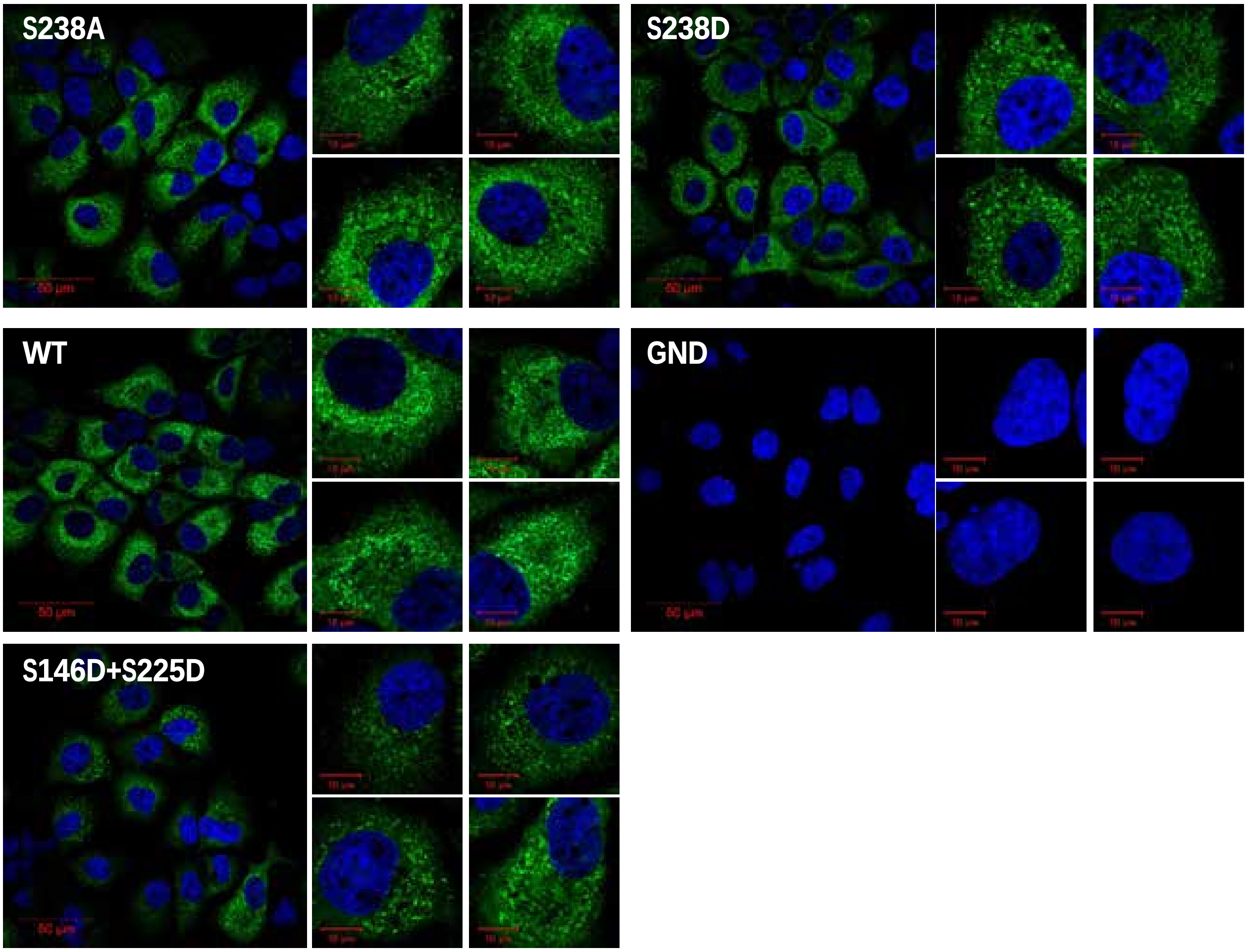


**Supplementary Figure 1c. Sub-cellular distribution of wildtype and serine mutant NS5A in JFH-1 infected cells.** Huh7 cells were electroporated with *in vitro* transcripts of either wildtype, NS5B GND (non-replicating) or the indicated NS5A mutant JFH-1 viruses. Cells were seeded onto coverslips and incubated for 96 h prior to fixation, immunostaining for NS5A (sheep, 1:1000) and imaging by confocal microscopy.

.

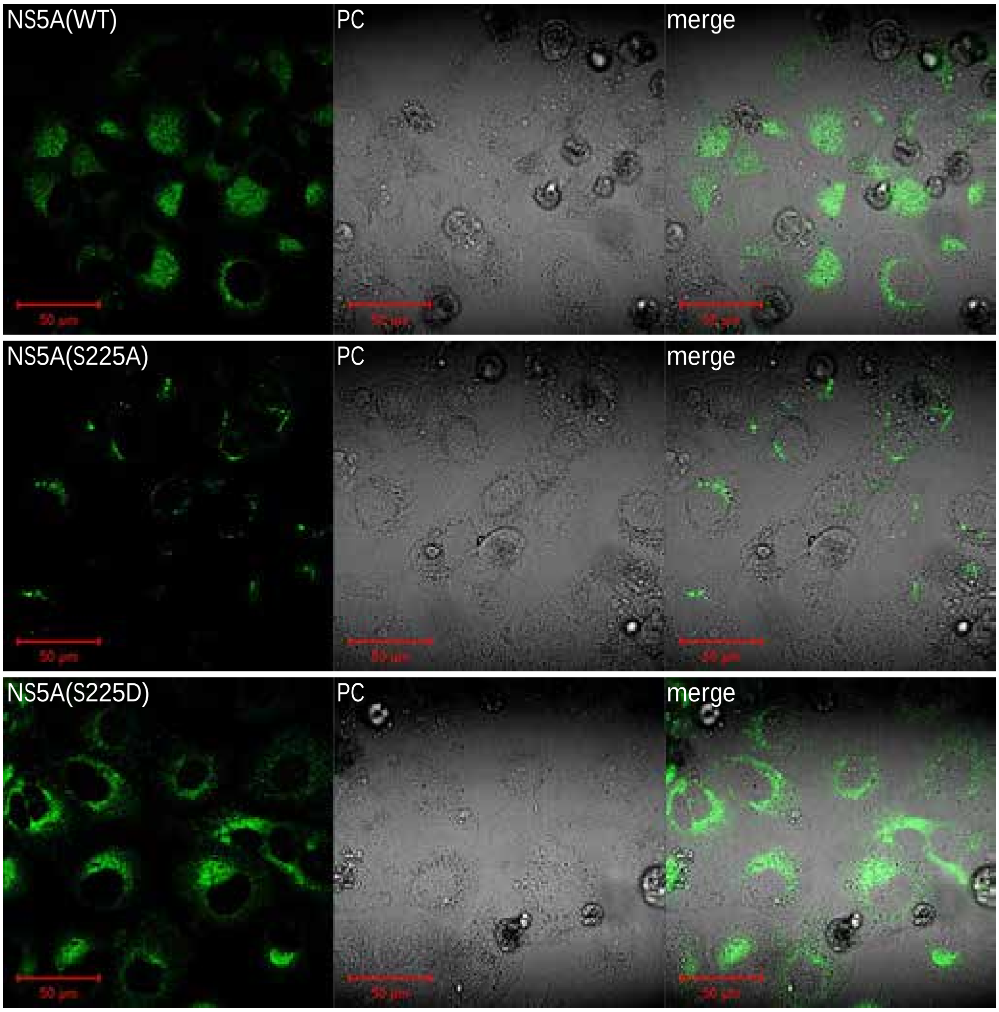


**Supplementary Figure 2. Widefield images of Huh7 cells infected with JcI-NS5A-emGFP viruses used for subsequent video capture.**

The two mutations S225A and S225D were generated in the JcI-emGFP virus construct (1) by conventional site-directed mutagenesis. Huh7 cells were electroporated with wildtype JcI-NS5A-emGFP, JcI-NS5A(S225A)-emGFP or JcI-NS5A(S225D)-emGFP RNA, seeded into 35mm glass bottom dishes and at 96 hpe imaged at 1 sec intervals on a Zeiss LSM 700 with environmental chamber. The videos were replayed at 20 fps, so are x 20 real time. PC, phase contrast.

(1) **Schaller, T., N. Appel, G. Koutsoudakis, S. Kallis, V. Lohmann, T. Pietschmann, and R. Bartenschlager. 2007.** Analysis of hepatitis C virus superinfection exclusion by using novel fluorochrome gene-tagged viral genomes. J.Virol. **81**:4591-603
